# Wound imaging software and digital platform to assist review of surgical wounds using patient smartphones: The development and evaluation of artificial intelligence (WISDOM AI study)

**DOI:** 10.1371/journal.pone.0315384

**Published:** 2024-12-09

**Authors:** Melissa Rochon, Judith Tanner, James Jurkiewicz, Jacqueline Beckhelling, Akuha Aondoakaa, Keith Wilson, Luxmi Dhoonmoon, Max Underwood, Lara Mason, Roy Harris, Karen Cariaga

**Affiliations:** 1 Guy’s and St Thomas’ NHS Foundation Trust, London, United Kingdom; 2 University of Nottingham, Nottingham, United Kingdom; 3 Islacare Ltd, London, United Kingdom; 4 University Hospitals of Derby and Burton NHS Foundation Trust, Derby, United Kingdom; 5 Liverpool Heart and Chest Hospital NHS Foundation Trust, Liverpool, United Kingdom; 6 Central and North West London NHS Foundation Trust, London, United Kingdom; University Magna Graecia of Catanzaro, ITALY

## Abstract

**Introduction:**

Surgical patients frequently experience post-operative complications at home. Digital remote monitoring of surgical wounds via image-based systems has emerged as a promising solution for early detection and intervention. However, the increased clinician workload from reviewing patient-submitted images presents a challenge. This study utilises artificial intelligence (AI) to prioritise surgical wound images for clinician review, aiming to efficiently manage workload.

**Methods and analysis:**

Conducted from September 2023 to March 2024, the study phases included compiling a training dataset of 37,974 images, creating a testing set of 3,634 images, developing an AI algorithm using ’You Only Look Once’ models, and conducting prospective tests compared against clinical nurse specialists’ evaluations. The primary objective was to validate the AI’s sensitivity in prioritising wound reviews, alongside assessing intra-rater reliability. Secondary objectives focused on specificity, positive predictive value (PPV), and negative predictive value (NPV) for various wound features.

**Results:**

The AI demonstrated a sensitivity of 89%, exceeding the target of 85% and proving effective in identifying cases requiring priority review. Intra-rater reliability was perfect, achieving 100% consistency in repeated assessments. Observations indicated variations in detecting wound characteristics across different skin tones; sensitivity was notably lower for incisional separation and discolouration in darker skin tones. Specificity remained high overall, with some results favouring darker skin tones. The NPV were similar for both light and dark skin tones. However, the NPV was slightly higher for dark skin tones at 95% (95% CI: 93%-97%) compared to 91% (95% CI: 87%-92%) for light skin tones. Both PPV and NPV varied, especially in identifying sutures or staples, indicating areas needing further refinement to ensure equitable accuracy.

**Conclusion:**

The AI algorithm not only met but surpassed the expected sensitivity for identifying priority cases, showing high reliability. Nonetheless, the disparities in performance across skin tones, especially in recognising certain wound characteristics like discolouration or incisional separation, underline the need for ongoing training and adaptation of the AI to ensure fairness and effectiveness across diverse patient groups.

## Introduction

Each year approximately 2.1 million surgical patients in England have wound healing problems after surgery, of which 500,000 become infected. Most of the complications happen after patients have been discharged from hospital, and this is set to increase with the increasing trend towards day surgery and early recovery from surgery programmes where patients are discharged as soon as safely possible [1].

Remote surgical wound monitoring, also known as postoperative wound monitoring or image-based monitoring, is a system where patients submit images and information about their wounds in response to an SMS text message or email request. The information submitted remotely by the patient is then reviewed by experienced clinicians [2]. Remote wound monitoring enables clinicians to review patients’ surgical wounds regularly and quickly after they have been discharged from hospital. Detecting complications in their developing stages enables wounds to be managed before they worsen and become harder and more expensive to treat. The benefits of remote wound monitoring include improved patient experience, fewer readmissions and re-operations, reduced mortality from surgical site infections (SSIs) and reduced demands on healthcare services [3,4].

Implementing a wound monitoring protocol faces practical challenges, including provider-level issues such as securing buy-in, and system-level challenges such as disrupting existing clinical workflows and integrating mHealth information into medical records [5]. A major drawback of digital remote monitoring systems is the additional workload for healthcare staff to quickly review incoming data [2]. For example, one heart surgery hospital receives 300 images each week to review [6]. Quick reviews are vital for early detection and management of issues like infections which is especially hard outside research settings due to limited surveillance resources [7]. One research study found that nearly one in ten patients had their submissions reviewed after more than 24 hours [8]. For this new service to work best, images need to be reviewed as soon as they are submitted. To be able to roll-out digital wound monitoring to the millions of patients having surgery in England, a system is needed where clinicians can manage this new workload quickly and efficiently.

To tackle these problems, artificial intelligence (AI) technologies can be used to make remote surgical wound monitoring more efficient and effective. AI algorithms can automatically analyse patient data, such as images, to spot signs of emerging conditions such as infections [9]. AI-driven image recognition can detect visual signs of dehiscence and inflammation, helping to sort and prioritise cases. This ensures that urgent cases are reviewed first by clinicians, reducing the workload on healthcare staff and improving patient care.

We have developed an AI algorithm that identifies surgical wound images requiring priority review by a clinician. This appears to be the first time AI has been used to examine surgical wound images to assist remote SSI monitoring. In 2022, a systematic review found no surgical wound monitoring technologies that were using AI to examine images for SSI monitoring or diagnosis [10], although a recent study using deep learning to distinguish common surgical complications demonstrated the potential for machine learning in this area [11].

The aim of this study was to develop and test an AI algorithm to identify surgical wound images which require priority review by a clinician.

## Methods and analysis

### Overview

There were three phases to the study which were conducted between September 2023 and March 2024. The first phase was the preparatory phase which included creating and annotating a training dataset and also creating a testing dataset. The training dataset included 37,974 unique patient images to build the AI algorithm. The testing dataset included 3,634 images to test the built AI algorithm, with a subset of 355 images which were used to assess the intra-rater reliability of the AI algorithm. The AI algorithm was built in the second phase and was designed to identify surgical wounds which required priority review by a clinician. In the third phase, the AI algorithm was tested for sensitivity, specificity and intra-rater reliability, overall and across different skin tones.

Patient and Public Involvement and Engagement (PPIE) and Equality, Diversity and Inclusion (EDI) representatives have been involved with this study in its design, funding acquisition, analysis and dissemination.

### Primary objectives

1. To assess whether AI can identify surgical wounds requiring either priority review or routine review with acceptable sensitivity compared to nurse specialists in surveillance.
2. To assess intra-rater reliability of the AI algorithm

### Secondary objectives

1. To assess the sensitivity, specificity, positive predictive value and negative predictive value of the algorithm’s ability to detect surgical wounds requiring priority review in light and dark and overall skin tones.
2. To assess specificity, positive predictive value and negative predictive value of the algorithm’s ability to detect wounds requiring priority review over all skin tones.
3. To assess the sensitivity, specificity, positive predictive value and negative predictive value of the algorithm’s ability to detect specific wound healing problems (e.g. discolouration) in light and dark skin.

### Sample sizes for testing datasets

To test primary outcome 1, based on 85% estimated sensitivity (with confidence interval +/- 3%) and a wound complication rate of 15%, 3,634 images were required.

To test primary outcome 2, to determine intra-rater reliability for wounds needing priority review, in light and dark skin, with a Kappa statistic 0.90 with 95% confidence interval +/– 0.05%, we required 355 images to be reviewed twice by the AI algorithm.

### Phase one: Preparing the datasets

#### Images used in the training dataset and the testing dataset

The images used to create the datasets to build and test the AI algorithm were held in an image library owned by Isla Care Ltd. Images were stored in the Google Cloud Platform (GoCP) via the Isla software platform in a web browser, encrypted via Secure Hash Algorithm (SHA)-256. The image library comprised 41,608 digital images of wounds which had been monitored remotely as part of adult patients’ routine care, using Isla software. The AI module is intended to be applicable to a wide range of surgical wounds. Consistency among photos was not required as the algorithm identifies ‘priority flags’ on standalone images, although patients were provided with advice on taking a usable photo. The images in the library therefore included ‘real-world’ images.

Images were sourced, stored and maintained under Data Protection Impact Assessment and Data Sharing Agreements. All stored images were anonymised by a clinician, for example, by cropping images to remove faces, tattoos, scars or background items. Images that the clinicians considered to be of insufficient quality for clinical decision making or that could not be edited for anonymization were excluded. No demographic data was collected and images from any patients who were opt-out registered were excluded. Remaining images were assigned a random ID code generated by the image library at the point of saving [12]. Authors with access to identifiable information adhered strictly to these protocols to protect participant confidentiality during and after data collection. Data security measures remained in place throughout the study duration and beyond, ensuring that participant privacy was upheld at all stages of the research process.

Eighty-seven percent (36,317) of the images in the training library were patient-captured and the remaining 13% (5,291) were clinician-captured. Images were obtained using different equipment, calibration, and lighting conditions. Variation in lighting and devices enables resultant models to be robust in real-world conditions. Models trained under consistent lighting or using specific devices may struggle to generalize to images captured under different lighting conditions [13]. By incorporating variations in lighting and device types during the training phase, the models become more adaptable to real-world scenarios, enhancing detection accuracy across diverse environments [14]. Approximately half the images obtained by clinicians were baseline images (i.e. images of healthy wounds obtained as part of a photo at discharge scheme) [15].

On 31 August 2023, a total of 3,634 (as per sample size calculations) were randomly selected for the study from the image library to create the testing database for sensitivity analysis. None of these images had been used to train the device, this sample of images was exclusively for assessing the sensitivity and specificity of the AI module. As per the sample calculations, a subset of 355 of the 3,634 images were randomly selected from the sensitivity analysis database to test for intra-rater reliability. The remaining 37,974 images were used as the training database to build the AI algorithm.

#### Preparing the training dataset to develop the algorithm

The dataset to build the algorithm contained 37,974 images of surgical wounds. Training dataset images continued to be stored on the Google Cloud Platform (GoCP) for security.

Each image was reviewed and bounding boxes (used to define the position and size of an object within an image) and annotations were applied. Images were annotated with one or more labels, depending on the features present. Labels followed a standard format outlined in the locally developed online data dictionary and described healing and non-healing wound features including necrotic tissue, slough, granulation tissue, bleeding, purulent exudate, serous exudate and also wound closure materials. Labels were applied individually to each image rather than in groups. This method was especially helpful for separate reviews, where less common examples were examined on their own.

Annotation was carried out by trained medical students using data in V7. Medical students were trained using the data dictionary and a test batch of images. Once students could annotate test batch images to a satisfactory standard they were allowed to annotate training dataset images.

Finally, two clinical nurse specialists and a machine learning developer conducted a final review of each annotation. Images that were approved were staged for future use. Images that were rejected were sent back with feedback for re-labelling by the medical student, and the review process was repeated. No images were deleted.

#### Preparing the testing dataset to test the algorithm (sensitivity analysis and inter-rater reliability)

The dataset to test the sensitivity analysis comprised 3,364 images randomly selected from the image library as per the sample size calculation. All random selections were undertaken by the trial statistician using Stata version 18 (a statistical analysis package). Testing dataset images continued to be stored on the Google Cloud Platform (GoCP) for security.

The randomisation was done in Stata version 18. Proprietary Stata commands to randomly select the patients/clinicians captured images to include in the sample and then to randomly select one image per patient/clinician. Firstly, a random selection of 3,634 patients was made, so that each patient was only represented once in the sample. If several images came from one patient, a single image was randomly selected to ensure only one image from each patient was included in the selected sample. Patients selected for the sample then had any additional images excluded from the training datasets as well as from the testing ones. Image IDs were checked to ensure there were no duplications. Some images that were not surgical wounds were found, these were replaced using a second randomisation that followed the same process as the original process, with the additional step of excluding any patient who had previously been selected.

A further sub sample of 355 images was selected randomly from the testing sample of 3,634 surgical wound images using Stata. The sub sample of 355 images was to test the AI algorithm for intra-rater reliability.

#### Categorising the testing dataset by skin tone

Skin tone labels were assigned to the 3,634 training dataset images manually by the specialist nurse to facilitate analysis by skin tone. Two thousand five hundred and ninety-two of the of the 3,634 images were from patients with light coloured skin (71%) and 1,042 were from patients with dark coloured skin (29%). The Fitzpatrick Application Programming Interface (API) library (derm-ita · PyPI) was installed to classify wound images according to the Fitzpatrick Scale skin tone model. Skin tone was calculated using the Individual Typology Angle (ITA) which is determined by the average of all pixel-wise ITA values extracted from skin images, taking into account the lightness and yellow-blue tints [16]. Generally, the ITA is calculated using the following the formula:

$$\mathrm{ITA}=\arctan\left(\frac{L-50}{b}\right)\times \left(\frac{180}{\pi }\right)$$

Here, L represents the luminance, and b represents the yellowness-blueness in the CIELAB color space. The ITA angle is expressed in degrees. Light skin tone is typically characterised by higher ITA values and might be defined as having an ITA greater than 55 degrees. This range indicates skin that reflects more light and thus appears lighter. Conversely, dark skin tones can be defined as having an ITA of less than 28 degrees (eg. skin that reflects less light, appearing darker).

For the purposes of this study, the following Fitzpatrick tones were classed at ‘light’ (Very light, Light 1, Light 2, Intermediate 1 and Intermediate 2) and the following Fitzpatrick skin tones were classed as ‘dark’ (Tan 1, Tan 2 and Dark). The skin tone classification was manually reviewed by a clinical nurse specialist and amended if necessary.

### Phase two: Training the AI algorithm

The AI algorithm was developed to identify images that contained at least one of the following non-healing or suture material features; discolouration, unexpected fluids/tissue, sutures/clips observed, incisional separation. Images that contained at least one feature identified by the AI algorithm were highlighted by the algorithm as requiring priority, or urgent, review by a clinician and were marked with a red flag on the Isla digital wound monitoring clinician review page.

The AI algorithm was built using classification and detection machine learning models with the training database which comprised 37,974 images. A ‘You Only Look Once‘ (YOLO) model, based on the open-source available from Ultralytics (https://docs.ultralytics.com/models/), was employed initially to provide real-time object detection to identify and locate the wound region of interest and the region of any drain sites or other incisions. YOLO is often chosen over traditional Convolutional Neural Networks (CNN)-based object detection models in clinical settings for its real-time processing capabilities, high accuracy and efficiency. While CNNs can capture object details at multiple scales, improving detection of various objects [17], YOLO’s unified detection framework provides a significant speed advantage, essential for timely healthcare decisions. YOLO balances accuracy and speed, understands the global context of images and operates efficiently on limited resources making it suitable for mobile and edge devices [18]. Although YOLO requires significant computational power, this is managed with scalable cloud computing that adjusts to demand. Additionally, YOLO’s need for a high volume of quality data is a common requirement for all machine learning frameworks and is therefore accepted.

To enhance overall accuracy and ensure system modularity for application in various use cases, models were trained on individual non-healing/infection features to guarantee sensitivity to all features, considering their varying occurrence rates across the feature set. Confidence thresholding was applied to filter out regions with lower confidence scores, along with region of interest (ROI) thresholding, where detections outside the specified main wound region of interest were discarded. This process was executed using a comprehensive validation set with a broad range of features and image types, representative of a ‘real-world setting’.

The algorithm combined several AI models for both image segmentation (e.g., to identify the wound in the image or closure materials like surgical clips) and classification (e.g., to classify between wounds with incisional separation and those with no incisional separation). The model outcomes were aggregated with logic to optimise performance, e.g., filtering out indications of dried blood where the indication wasn’t significant in comparison to the size of the wound. This logic aggregated all model results into a binary outcome for each image, ‘priority’ or ‘routine’ review, based on whether one or more of the wound healing descriptors were present (discolouration, unexpected fluids/tissue, sutures/clips observed, and incisional separation). These four features were chosen because these elements allow the AI to comprehensively monitor and analyse key visual indicators of wound healing, providing valuable insights into potential complications (in future clinical evaluations, sutures/clips would only flag if more than fourteen days from operation). This processing was by a script to automatically sequence all images through the algorithm and generate the set of results.

### Phase three: Testing

#### Testing the AI algorithm

The performance of the AI algorithm was tested against clinical nurse specialists’ assessments using the testing dataset of 3,634 images for sensitivity of wound features that required a priority review and also across light and dark skin tones, with an additional assessment for intra-rater reliability on 355 images.

Standard practice has nurse specialists in wound surveillance or wound healing reviewing wounds to assess healing. Therefore, review by a specialist nurse was taken to be the ‘gold standard’ against which the AI algorithm was assessed. The testing dataset of 3,634 images was reviewed independently by both the AI algorithm and specialist nurses. The specialist nurses were trained using the same data dictionary and test batch of images used for the AI training. This training ensured consistency in evaluation criteria, facilitating a clear and justified comparison between human and AI assessments. The binary outcome of ‘requires priority review’ from the AI algorithm and the specialist nurses was compared to calculate the accuracy of the AI algorithm. The outcome of requiring a priority review was determined by an image containing at least one of the following four features (discolouration, unexpected fluids/tissue, sutures/clips observed, and incisional separation).

Each image was reviewed by two specialist nurses independently to determine its outcome. Where there was disagreement regarding the outcome, a third specialist nurse was consulted to provide the deciding outcome.

All 3,634 images within the testing dataset were passed through the AI algorithm to test for sensitivity.

The AI algorithm was tested for intra-rater reliability to confirm that the algorithm reaches the same outcome when presented with the same image on more than one occasion. The subset of 355 randomly selected images from the testing database, described in ‘Preparing the Dataset’ were passed through the algorithm a second time. The outcome (priority review or no priority review) calculated by the AI algorithm for the first pass of each image was compared against the outcome from the AI algorithm for the second pass of each repeated image.

### Data analysis

The primary outcome measure for objective 1 was sensitivity of wounds needing priority review versus wounds needing routine review compared with the reference standard of the clinical assessments. Sensitivity was defined as the proportion of wound images correctly identified as ‘priority review’ compared with the “gold standard”. Sensitivity is of higher importance for this study because it is preferred for the algorithm to identify healing wounds as ‘priority review’ than to miss a wound that needs a priority review. The sensitivity of detecting wounds with healing problems is reported as a percentage, with 95% confidence intervals. Sensitivity is measured using the following:

$$Sensitivity=\frac{True\;Positives}{\left(True\;Positives+False\;Negatives\right)}\times 100$$

Primary outcome measure 2, intra-rater reliability, is reported as the number and percentage of concordant ratings (when the two assessments of each pair of images agree) and discordant assessments (when the two assessments of each pair of images disagree). Intra-rater variance will be assessed using the kappa statistic (with a 95% confidence interval). The Kappa statistic can range in value from minus one (where a negative value would indicate the AI algorithm was performing worse than if it had made the assessments randomly, and a score of minus one would indicate that all of the identical images were rated differently by the AI algorithm) up to 1 (if it rates all of the duplicate images exactly the same both times). The AI algorithm has been trained to identify wound healing problems and to assess whether wounds need a priority review of not. However, it is not a dynamic AI algorithm, so the assessment process will not change in response to the first time it assessed the duplicate images. The process also has no "memory", so it will not "remember" the assessments it made the first time it assessed the images, therefore the two assessments will be independent of each other.

Percentages and 95% confidence intervals were used to report the following secondary outcomes:

- The sensitivity of detecting wounds requiring priority review specifically in dark and light skin tones.
- The specificity, positive predictive value (PPV) and negative predictive value (NPV) of detecting priority review in dark skin tones, light skin tones and over all skin tones.
- The sensitivity, specificity, positive predictive value (PPV) and negative predictive value (NPV) of detecting suture/staples, discolouration/redness, unexpected fluid or tissue and separation of the incision in dark skin tones, light skin tones and over all skin tones (using ATI skin tone, with human assessor).

Specificity defines what percentage of wounds requiring routine reviews were correctly identified as not requiring priority review by the algorithm.


$$Specificity=\frac{TrueNegatives}{\left(TrueNegatives+FalsePositives\right)}\times 100$$

PPV defines the percentage of images with a positive result (i.e. priority review) that had a wound healing problem.


$$PPV=\frac{True\;Positives}{\left(True\;Positives+False\;Positives\right)}\times 100$$

NPV defines the percentage of images with a negative result (i.e. routine review) that did not have a wound healing problem.


$$NPV=\frac{True\;Negatives}{\left(True\;Negatives+False\;Negatives\right)}\times 100$$

## Results

### Primary outcomes

#### Sensitivity of identifying wound images that need a priority review

The sensitivity of the AI algorithm was 89% (95% confidence interval: 87% to 91%) which exceeds the target sensitivity specified in the protocol, which was 85% with a 95% confidence interval of +/- 3%. See Table 1.

**Table 1:** Primary outcome 1—Sensitivity of need for priority review over all skin tones.

PRIMARY ANALYSIS sensitivity of needing a priority review	Clinician (Gold standard) assessment		Total assessments
AI assessment	Priority review	standard review	
**Priority Review**	1,014 (89)	1,073 (43)	**2,087 (57)**
**Standard review**	122 (11)	1,425 (57)	**1,547 (43)**
**Total assessments**	**1,136 (100)**	**2,498 (100)**	**3,634 (100)**
**Sensitivity % (95% CI)**	**89.26 (87.31 to 91.00)**		

### Intra-rater reliability of AI algorithm: Identifying wound images that need a priority review

The AI algorithm rated the subset of 355 images that were presented to the algorithm twice for prioritisation. The AI algorithm rated each image identically both times, therefore Kappa = 1. As the images were rated with 100% agreement, a 95% confidence interval for Kappa cannot be calculated. See Table 2.

**Table 2:** Primary outcome 2—Intra-rater reliability.

PRIMARY ANALYSIS Intra-rater reliability: Kappa statistic	First assessment			Total assessments
Second assessment	Priority review	No priority review / standard review		
**Priority Review** n (%)	210	0		**210 (59)**
**No priority review / standard review** n (%)	0	145		**145 (41)**
**Total assessments**	**210 (100)**	**145 (100)**		**355 (100)**
**Concordant and discordant assessments %** **(95% CI)**	**Concordant images**		**Discordant images**	
	**100 (98.97 to 100)** ^**a**^		**0 (0 to 1.03)** ^**b**^	
**Kappa statistic**	**1**			
**SECONDARY ANALYSIS** **Intra-rater reliability: probability of a chance error in assessment**	**0**			

^a^ As there is 100% agreement, this is the one sided 97.5% confidence interval, showing the lower bound for the percentage of agreement.

^b^ As there is 0% disagreement, this is the one sided 97.5% confidence interval, showing the upper bound for the percentage of disagreement.

### Secondary outcomes

#### Sensitivity in light and dark skin tones

There were disparities in the AI algorithm’s ability to detect discolouration, sutures or staples and incisional separation in light and dark skin tones (Table 3). The biggest difference in sensitivity was for incisional separation. In light skin tones, the AI algorithm correctly identified 70% of the wounds with incisional separation (95% Confidence Interval (CI): 55% to 83%), however in dark skin tones it correctly identified only 48% (95% CI: 25% to 70%), a difference of 22%. With regard to redness and discolouration, and sutures or staples, there was a disparity of 6% between the sensitivity of their detection in light skin compared to dark. Although the sensitivity of detecting sutures or staples was 80% in people with dark skin, but sensitivity of detecting redness or discolouration was only 48%. However, the sensitivity of identifying images that needed a priority review were very similar in people with light and dark skin tones.

**Table 3:** Secondary outcomes.

SECONDARY ANALYSIS	Sensitivity (95% CI)	Specificity % (95% CI)	PPV (95% CI)	NPV (95% CI)
**Sutures or staples (%)**				
Light skin tones (n* = 682) Dark skin tones (n = 166) All skin tones (n = 848)	*86 (83 to 89)* *80 (73 to 85)* *85 (82 to 87)*	*86 (84 to 87)* *84 (81 to 86)* *85 (84 to 86)*	*68 (65 to 71)* *49 (42 to 55)* *63 (61 to 66)*	*95 (93 to 96)* *96 (94 to 97)* *95 (94 to 96)*
**Discoloration or redness (%)**				
Light skin tones (n = 195) Dark skin tones (n = 31) All skin tones (n = 226)	*64 (56 to 70)* *48 (30 to 67)* *62 (55 to 68)*	*77 (75 to 78)* *86 (84 to 88)* *80 (78 to 81)*	*18 (15 to 21)* *10 (6 to 16)* *17 (14 to 19)*	*96 (95 to 97)* *98 (97 to 99)* *97 (96 to 98)*
**Incisional separation (%)**				
Light skin tones (n = 47) Dark skin tones (n = 21) All skin tones (n = 68)	*70 (55 to 83)* *48 (26 to 70)* *63 (51 to75)*	*87 (86 to 89)* *89 (87 to 91)* *88 (87 to 89)*	*9 (6 to 13)* *8 (4 to 15)* *9 (7 to 12)*	*99 (99 to 100)* *99 (98 to 99)* *99 (99 to 99)*
**Unexpected fluids or tissue (%)**				
Light skin tones (n = 154) Dark skin tones (n = 47) All skin tones (n = 201)	*73 (66 to 80)* *70 (55 to 83)* *73 (66 to79)*	*82 (81 to 84)* *87 (85 to 89)* *84 (83 to 85)*	*21 (17 to 24)* *21 (15 to 28)* *21 (18 to 24)*	*98 (97 to 99)* *98 (97 to 99)* *98 (98 to 99)*
**Urgent review (%)**				
Light skin tones (n = 908) Dark skin tones (n = 228) All skin tones (n = 1,136)	*89 (87 to 92)* *88 (83 to 92)* *89 (87 to 91)*	*54 (51 to 56)* *64 (60 to 67)* *57 (55 to 59)*	*51 (49 to 54)* *40 (36 to 45)* *49 (46 to 51)*	*91 (87 to 92)* *95 (93 to 97)* *92 (91 to 93)*

n = the number of wound attributes (e.g. sutures, discoloration/redness etc.) observed by the clinicians.

#### Specificity in light and dark skin tones

Specificity calculates the percentage of images without a problem that the AI algorithm correctly identified as not having a problem. For discolouration/redness, the specificity was better for people with dark skin tones. Over four-fifths (86%, 95% CI: 84% to 88%) of wounds without problems in people with dark skin tones were correctly identified compared to 77% (95% CI: 75% to 78%) correctly identified in people with light skin tones. To some extent this might be expected because as sensitivity decreases, specificity increases.

The other wound healing aspect with a relatively large discrepancy in specificity was identifying wounds that needed a priority review. Nearly two-thirds of wound images from people with dark skin tones were correctly identified as not needing a priority review (64%, 95% CI: 60% to 67%), compared with 54% (95% CI: 51% to 56%) of images from people with light skin tones.

#### Positive predictive value (PPV) in light and dark skin tones

The Positive Predictive Value shows the probability (expressed as a percentage) that an image the AI algorithm identified as needing a priority review really needed one. This has to be interpreted cautiously in this study as images from a variety of operations were included in the analysis dataset and it is possible that the positive predictive value could vary by operation type. However, with regard to light and dark skin tones, there was one marked difference which was in the PPV for sutures or staples. In the images from patients with light skin tones there was a 68% probability that an image flagged by the AI algorithm as having sutures/staples really did have them (95% CI: 65% to 71%). In the images from patients with dark skin tones, there was only a 49% probability that the images truly showed staples or sutures (95% CI: 42% to 55%). The probability that patients that were identified by the AI algorithm as having redness or discolouration that really did was lower for patents with dark skin tones (10%, with 95% confidence interval: 6% to 16%) than patients with light skin tones (18%, with 95% confidence interval 15% to 21%).

#### Negative predictive value (NPV) in light and dark skin tones

The Negative Predictive Value shows the probability (expressed as a percentage) that an image the AI algorithm identified as not needing a priority review really did not need one. This has to be interpreted cautiously in this study for the same reason as for the Positive Predictive Value. The negative predictive values in patients with light and dark skin tones were very similar, although patients with dark skin tones had a slightly higher NPV for identifying patients who did not need a priority review (95% with 95% confidence interval: 93% to 97%) compared to 91% (with 95% confidence interval: 87% to 92%) for patients with light skin tones.

#### Clinicians’ observations of wound attributes

See Table 4. Overall, 2,592 of the 3,634 images on the testing dataset were from patients with light coloured skin (71%) and 1,042 were from patients with dark coloured skin (29%). If wound problems were observed by clinicians with equal frequency in images from patients with light and dark coloured skin, approximately 71% of each wound problem would be observed in images from patients with light coloured skin and 29% from images of patients with dark coloured skin. However, this was not always the case: 86% of the cases of redness and discolouration were observed in patients with light coloured skin and only 14% were observed in patients with dark coloured skin. Similarly, 80% of the sutures were observed in images from patients with light coloured skin and 20% in patients with dark coloured skin and for unexpected fluids 77% of cases were observed in patients with light coloured skin and 23% in patients with dark coloured skin. The observations of incisional separation were similar to the expected percentages at 69% in patients with light coloured skin and 31% in patients with dark coloured skin. Overall, 80% of priority reviews were identified as needed by patients with light coloured skin and 20% needed by patients with dark coloured skin. Possibly patients with dark coloured skin are less likely to display these signs of wound problems, or they are more difficult to observe in images from people with dark coloured skin.

**Table 4:** Wound healing problems observed by clinicians.

SECONDARY ANALYSIS	Number of images with the problem	Percentage of images with the problem
**Sutures or staples**		
Light skin tones Dark skin tones All skin tones	*682* *166* *848*	*80* *20* *100*
**Discoloration or redness**		
Light skin tones Dark skin tones All skin tones	*195* *31* *266*	*86* *14* *100*
**Incisional separation**		
Light skin tones Dark skin tones All skin tones	*47* *21* *68*	*69* *31* *100*
**Unexpected fluids or tissue (%)**		
Light skin tones Dark skin tones All skin tones	*154* *47* *201*	*7* *23* *100*
**Urgent review (%)**		
Light skin tones Dark skin tones All skin tones	*908* *228* *1136*	*80* *20* *100*

## Discussion

The advent of remote surgical wound monitoring presents a significant opportunity to enhance post-operative care, particularly as the prevalence of wound healing issues continues to rise [19], compounded by the growing trend towards day surgery and early recovery programs. Our study aimed to validate an AI algorithm designed to identify surgical wound images necessitating priority review, thus potentially streamlining the management of post-operative complications in a scalable manner. Our findings demonstrate promising results, with the AI algorithm exhibiting a sensitivity of 89% in identifying wounds requiring priority review, surpassing our predefined target sensitivity of 85%. This underscores the algorithm’s potential to effectively triage wound images and alert clinicians to cases needing immediate attention, thereby mitigating the risk of complications progressing unnoticed.

A previous study [11] used Convolutional Neural Networks (CNNs) to detect early surgical complications. Our study employs YOLO models for its AI algorithm development. Although CNNs may have better accuracy in detecting small objects, more precise localisation, and superior handling of fine-grained details compared to YOLO object detection methods [20], we chose this form of deep learning architecture because of its superiority for real-time use. YOLO is faster than traditional CNNs because it only needs to look at the picture once to make predictions, considering the whole image rather than just parts of it. It also combines detecting, locating, and classifying objects all at once, which makes it more efficient. Unlike traditional CNNs, YOLO is better at predicting where objects are in the image, so it makes fewer mistakes [20]. A further distinction of this work is the inclusion of skin tone in the analysis.

In our study, both clinicians and the AI algorithm noticed differences in the presentation of wound problems based on skin tone. For instance, both observed that certain wound issues like redness and discolouration were more prevalent in patients with light skin tones compared to those with dark skin tones. Clinicians tended to observe certain wound attributes, such as redness and sutures, more frequently in patients with light skin tones compared to dark skin tones. In contrast, the AI algorithm showed differences in sensitivity and specificity based on skin tone, with variations in correctly identifying wound problems. This highlights potential areas for improvement in algorithmic performance and mitigating biases (such as observation bias) in healthcare AI systems.

Our study confirms the intra-rater reliability of the AI algorithm, with a perfect agreement (Kappa = 1) observed in identifying wound images necessitating priority review upon re-evaluation. This high level of consistency indicates the reliability and reproducibility of the AI algorithm’s assessments, essential for its integration into clinical practice.

However, disparities were observed in the AI’s ability to detect certain wound attributes across different skin tones. Redness and discolouration were more frequently observed in patients with light skin tones, suggesting potential challenges in detecting these issues in darker skin tones. Sensitivity for specific wound problems varied across skin tones, with lower sensitivity observed in detecting incisional separation and discolouration in dark skin tones. Specificity was generally high, with better performance in identifying wounds without problems in people with dark skin tones. However, there was a marked discrepancy in specificity in identifying wounds needing a priority review, with higher accuracy observed for images from people with dark skin tones. PPV varied across skin tones, particularly for identifying sutures or staples, indicating reduced accuracy in images from patients with dark skin tones. NPV was similar across skin tones, with a slightly higher NPV observed for images from patients with dark skin tones in identifying whether priority review was not needed. Several factors can contribute to this variability, including the diversity and representativeness of the dataset, the quality of images, and the specific wound features being identified. These findings underscore the importance of considering equity in AI development to ensure effectiveness across diverse patient populations.

The AI system’s high sensitivity (89%) and perfect intra-rater reliability suggest that it can reliably identify priority wound cases, allowing clinicians to focus on the most urgent cases first. This complementary role of AI and humans could help scale digital remote wound monitoring, leading to more efficient use of healthcare resources and potentially better patient outcomes by reducing unnecessary delays in treatment.

However, the study also highlighted disparities in the AI system’s performance across different skin tones, particularly in detecting discolouration and incisional separation. Addressing these disparities is crucial to ensure equitable care for all patients, regardless of their skin tone. As we continue to work with new, larger datasets, it is important to identify and address biases to further refine the AI algorithm [21].

These findings underscore the importance of ongoing evaluation and refinement of AI systems in clinical practice to ensure they contribute positively to patient outcomes. By improving the accuracy and fairness of AI tools, we can support clinicians more effectively and advance towards the strategic goals outlined in the NHS Long Term Plan [22].

We will continue to refine our algorithm and addressing the observed disparities in algorithm performance across diverse skin tones. Further research is warranted to address disparities and enhance the diagnostic accuracy of AI algorithms in remote wound monitoring, ultimately improving healthcare outcomes for all patients. There are several plausible future steps for improving the AI’s performance across different skin tones. More data representing dark skin tones is essential and can ideally be obtained through collaborations with other healthcare institutions, adding more diverse real images of skin colours to our existing database [23]. Accuracy could be significantly improved by developing specialist models, such as an ensemble of models tailored to different skin tones and leveraging transfer learning on diverse datasets [24]. This is a technique used in situations like this, but due to the lack of open-source data in general, we could not use this technique to improve sensitivity on darker skin tones. Training on images using colour profiles more suited to working with skin tones, such as CIELab [25], will allow the models to detect more subtle differences in colour, particularly on darker skin tones. Generating synthetic images using techniques like Generative Adversarial Networks (GANs) could boost data quantity, and has many advantages, including overcoming difficulties related to obtaining volume, labelling data, and privacy [26]. However, we are not currently exploring this option due to concerns over representativeness [27].

In this context, AI can ensure timely identification and intervention for wound issues by applying best practices. Our model shows potential in reducing inconsistencies in remote care and surveillance pathways [28], decreasing the risk of overtreatment [29], and assisting clinicians who are struggling to find time for submission reviews alongside their existing clinical duties [8]. However, to align with the NHS Long Term Plan’s [22] strategic goals, implementing this AI system in clinical practice needs careful evaluation to ensure its safe and ethical use on a large scale. Therefore, a subsequent study evaluating the AI will investigate potential implementation challenges, such as integration with clinical workflows, electronic health records, staff and patient acceptance, feasibility outcomes, safety outcomes, training, economic modelling and regulatory requirements for market readiness [30].

Further work is needed to understand and explain how the algorithm functions, ensuring it does not perpetuate or worsen existing biases in healthcare but instead reduces health inequalities and improves care outcomes. We will develop and integrate Explainable AI (XAI) techniques to make AI decisions interpretable. Methods like SHAP (SHapley Additive exPlanations) values and LIME (Local Interpretable Model-agnostic Explanations) will help clinicians and stakeholders understand AI predictions.

Implementation of AI in healthcare settings is at a very early stage [31]. A study by Nelson et al [32] found that patients were receptive of the use of AI in healthcare as long as it maintains the integrity of the clinician-patient relationship. It is important to point out that in the present study, the AI is not making a diagnosis of infection, the clinician still reviews and assesses all images.

A key limitation of this study was the use of an automated ITA-based skin tone estimation method, due to a lack of public datasets with skin tone and ethnicity. There are many risks associated with ITA including sensitivity to lighting conditions, non-skin imaging artefacts that can create misleading results, lesion-to-skin contrast, and errors from pixel to image-level decisions [16]. To mitigate these risks, we used human assessors to review and amend skin tone assessments, and although not ideal, we opted for a binary outcome (dark or light). Several factors contribute to the lower sensitivity in detecting wound characteristics in darker skin tones. In the training set, 64% of images were light skin tone and 36% were dark skin tone. This leads to a bias towards the majority class (lighter skin tone) because the model has not been adequately trained on a diverse set of examples. This issue should not be confused with an unbalanced dataset, as the data volume is too low to train a separate model for just dark skin tone data [33]. Wound features look different on dark skin tones, leading to many conflicts within our training data, resulting in poor labels that could affect sensitivity [34]. Notably, similar disparities were observed among human (nurse specialist) reviewers in the test dataset. Additionally, 2D imaging devices may show biases due to differences in light absorption and reflection on various skin tones, affecting image quality and accuracy [29,35]. Other studies on 3D AI in chronic wound measurement have also found accuracy issues with darker skin tones [36–38]. Clinician bias and a lack of equitable assessment across skin tones may also contribute to lower sensitivity. This can lead to complications being under-recognised in highly pigmented skin, potentially causing delayed or missed diagnoses and worse wound outcomes [39]. A suggestion for future studies is to assess skin tone face to face and not through images, which will permit more nuanced assessments of the effect of skin tone to be undertaken.

Under-representation of wound images taken by patients due to socio-economic status, advanced age, or other forms of technology exclusion may have been offset by our inclusion of images captured by clinicians in addition to patient captured images [40,41]. Nevertheless, the method of image collection in the image library which may have introduced some bias that could skew predictions or lead to inaccurate results from the algorithm. For example, images of patients under 18 years old were excluded from image library, thus the algorithm did not capture the diversity of wound healing across younger individuals, and age-related factors influencing wound healing could be underrepresented. Finally, eighty-seven percent (36,317) of the images in the training library were patient-captured and the remaining 13% (5,291) were clinician-captured. It is not possible to state the percentage of patient or clinician captured images within the AI analysis as the images were now anonymised. However, our robust randomisation should ensure adequate representation.

## Conclusion

In summary, our study highlights the potential of remote surgical wound monitoring facilitated by AI to enhance post-operative care in the face of increasing wound healing issues and outpatient procedures. Validating an AI algorithm designed to identify surgical wound images necessitating priority review yielded promising results, with the algorithm demonstrating a sensitivity of 89%, exceeding our predefined target and indicating its potential to efficiently triage wound images for timely intervention. Moreover, the algorithm’s intra-rater reliability was confirmed, showcasing its consistency and readiness for integration into clinical practice.

However, our findings also shed light on disparities in the algorithm’s performance across different skin tones, particularly in detecting specific wound attributes. Challenges in identifying issues such as redness and discolouration in darker skin tones highlight the importance of equitable AI development to ensure effectiveness across diverse patient populations. Addressing these disparities and further refining the algorithm’s diagnostic accuracy through ongoing research will be crucial for optimising remote wound monitoring and improving healthcare outcomes for all patients.
